# Durable Superhydrophobic Coatings on Tungsten Surface by Nanosecond Laser Ablation and Fluorooxysilane Modification

**DOI:** 10.3390/ma16010196

**Published:** 2022-12-26

**Authors:** Ekaterina A. Kuzina, Kirill A. Emelyanenko, Maria A. Teplonogova, Alexandre M. Emelyanenko, Ludmila B. Boinovich

**Affiliations:** 1 A.N. Frumkin Institute of Physical Chemistry and Electrochemistry, Leninsky prospect 31 bldg. 4, 119071 Moscow, Russia; 2 N.S. Kurnakov Institute of General and Inorganic Chemistry, Leninsky prospect 31, 119071 Moscow, Russia

**Keywords:** tungsten, superhydrophobic coatings, laser treatment, durability, sand abrasion

## Abstract

Tungsten is an attractive material for a variety of applications, from constructions in high-temperature vacuum furnaces to nontoxic shields for nuclear medicine, because of its distinctive properties, such as high thermal conductivity, high melting point, high hardness and high density. At the same time, the areas of the applicability of tungsten, to a large extent, are affected by the formation of surface oxides, which not only strongly reduce the mechanical properties, but are also prone to easily interacting with water. To alleviate this shortcoming, a series of superhydrophobic coatings for the tungsten surface was elaborated using the method of nanosecond laser treatment followed by chemical vapor deposition of hydrophobic fluorooxysilane molecules. It is shown that the durability of the fabricated coatings significantly depends on surface morphology and composition, which in turn can be effectively controlled by adjusting the parameters of the laser treatment. The coating prepared with optimized parameters had a contact angle of 172.1 ± 0.5° and roll-off angle of 1.5 ± 0.4°, and preserved their high superhydrophobic properties after being subjected to oscillated sand abrasion for 10 h, continuous contact with water droplets for more than 50 h, and to several cycles of the falling sand test.

## 1. Introduction

Tungsten is commonly used in both high temperature and room temperature applications. High thermal conductivity, high melting point, low sputtering yield and high plasma sputtering erosion resistance, high hardness and density make it a good choice for high-temperature materials used in vacuum resistance furnaces, bulb filaments, plasma-facing components [1,2,3]. At room temperatures, the high atomic number and high-density of tungsten allows considering it as the best nontoxic shielding material for nuclear medicine, where the small thickness of tungsten is required to shield the high energy of gamma radiation [4]. The areas of the applicability of tungsten, to a large extent, are affected by the formation of surface oxides which were successfully used in photocatalysis, electrochemistry, electrochromic devices, and phototherapy [5,6,7,8,9,10]. At the same time, tungsten trioxide, which is most often present in the tungsten surface, easily interacts with water [11,12]. The latter results in the formation of hydrates, whose structure is highly dependent on their water content [12]. Consequently, the state of the tungsten surface and its mechanical properties reveal to be highly sensitive to the contact with water, which can compromise some of the applications of tungsten as an engineering or functional material.

One of the most powerful routes to protect the surface of any material against interaction with aqueous media is to impart the superhydrophobic state to such a surface [13,14,15,16]. Superhydrophobic coatings, due to their unique properties and the peculiarities of interaction with aqueous media, are characterized by high corrosion resistance [14,17,18,19], icephobic properties [20,21,22,23], bactericidal properties [24,25,26,27,28], resistance to biofouling [27,28,29], and many others. To create stable superhydrophobic coatings on various substrates, three main requirements should be fulfilled. Namely, the surface of the material should have low surface energy, multimodal roughness, and, finally, the shape and size of the texture elements on the surface ensure the required contact angle [30]. The formation of the hierarchical surface roughness with micro- and nanoelements of the surface texture is a primary task, which can be solved by various methods [13,16,22,25]. Among these, the creation of surface roughness by laser pulses is a unique approach that has the advantages of non-contact, automated and direct fabrication [31,32]. This method makes it possible to increase the mechanical strength of the texture by changing the chemical and phase composition, as well as the structure of the material [33].

Although several papers reported the fabrication of superhydrophobic coatings on tungsten surfaces [34,35,36], little attention has been paid so far to the issue of the chemical stability and mechanical durability of obtained surfaces under exploitation loads. Therefore, the primary goal of this study was the development of methods for obtaining chemically and mechanically durable superhydrophobic coating on the tungsten surface. By adjusting the parameters of the nanosecond infrared pulsed laser treatment and consequent deposition of the hydrophobic fluorooxysilane layer, we prepared a series of superhydrophobic coatings and evaluated their exploitation potential with several chemical and mechanical durability tests. 

## 2. Materials and Methods

### 2.1. Sample Preparation

Industrial-grade tungsten plates (W content > 99.95%) with a thickness of 0.5 mm were cut into rectangular 15 × 20 mm^2^ samples. The samples were ultrasonically washed sequentially in distilled water and isopropyl alcohol to remove dust, dirt, and oil contaminations and dried in the air prior to the laser treatment. 

Nanosecond infrared pulsed laser radiation was used to create the surface texture with hierarchical roughness, which is one of the main prerequisites for obtaining the stable superhydrophobic state [20,21]. An infrared ytterbium fiber laser with a wavelength of 1.064 μm was used, with the possibility of varying the pulse duration in the range from 4 to 200 ns, pulse frequency from 20 to 100 kHz, and energy of the single pulse up to 0.95 mJ in the TEM_00_ mode. The laser beam was focused on the surface of the processed sample in the spot of approximately 40 μm in diameter and moved along the sample surface in two mutually perpendicular directions using a computer-controlled two-axis deflection system MS10 (Raylase, Germany). In the zone of action of the laser beam, the material was subjected to heating to a high temperature, melting and sublimation or explosive ablation, during which atoms of the material were removed from the surface and redeposited around the ablated zone in the form of micro- and nanoparticles. From previous experience, the most effective textures for different metals were obtained in the explosive ablation regimes [23,31,32,33]. Since tungsten is the most refractory metal, in order for explosive ablation of the material on the surface to occur, it was necessary to maximize the energy of the radiation incident on the sample. Therefore, the pulse duration of 200 ns and the corresponding repetition rate (pulse frequency) of 30 kHz were chosen. To obtain surfaces with different morphologies, the texturing mode was selected by changing the processing speed, the scanning pitch (distance between two neighboring laser paths), and the number of repetitive laser passes over each scanning line. For some texturing regimes (as will be rationalized below in Results and Discussion section), the samples were additionally heated with a help of an electric hotplate C-MAG HP 7 (IKA, Germany). The samples were placed on ceramic glass surface heated to 500 °C for 10 min prior to the laser treatment, and the plate was kept hot during the laser treatment.

After the laser texturing, the surface became superhydrophilic due to imparted multimodal hierarchical roughness [33]. The superhydrophilicity could be easily verified by the fast complete spreading of water droplet touching the surface. To reduce the surface energy, we used the deposition of a hydrophobic agent, methoxy-{3[(2,2,3,3,4,4,5,5,6,6,7,7,8,8,8-pentadecaflourooctyl)-oxy]-propyl}silane, synthesized in the laboratory of Prof. Muzafarov as described elsewhere [37]. Prior to hydrophobization, the laser-treated samples were ultrasonicated (35 kHz, 55 W) in distilled water for 10 min (Ultrasound bath Grad 13-35, LLC Grad-Technology, Russia) to remove from the surface the nanoparticles which were weakly adhered during the laser treatment. Then the samples were dried for 30 min at 150 °C in air oven and subjected for 20 min oxygen plasma treatment at oxygen pressure of 25±5 Pa and plasma power of ~10 W (Plasma Cleaner PCE-6, Zhengzhou CY Scientific Instrument, China). Such oxygen plasma treatment was proven earlier [38,39] to be an effective tool for enhancing the density of active chemisorption centers on the surface for properly binding the molecules of the hydrophobic agent. Immediately after the plasma treatment, the samples were placed into a hermetically sealed vessel with saturated vapors of the hydrophobic agent and kept for 1 h at a temperature of 105 °C. At this time, the hydrophobic agent was chemisorbed on the active centers of the micro- and nanotexture elements. After chemisorption of fluorooxysilane, the samples were washed in isopropyl alcohol and then in acetone to remove the molecules which were not bonded chemically but adsorbed physically. Next, the samples were dried in an oven at a temperature of 150 °C. At that stage, a layer of fluorooxysilane molecules was crosslinked by siloxane bonds to form a 2D polymer network enveloping the micro- and nanotexture obtained by the laser treatment.

### 2.2. Characterization and Testing

The wettability of samples was characterized by measuring the contact and roll-off angles for water droplets with a volume of 15 µl, using the custom-made experimental setups described earlier [23,40]. The deionized water was used as a test liquid. Each reported value represents an average of measurements made for at least 5 different positions on the substrate. As a criterion for primary selection of the laser treatment parameters, we considered the contact angles above 170° and roll-off angles below 5°. Such samples were subjected to further testing of chemical and mechanical durability. 

The chemical durability of the coating in contact with liquid water or water vapors was studied inside a double wall glass vessel [40], which makes it possible to maintain 100% atmospheric humidity around the sample. To study the resistance in contact with water, a drop of water was placed on the surface of the sample and the change in its contact angle, surface tension, contact diameter and volume was monitored over time. The experiment was carried out in an atmosphere of saturated water vapors, which was necessary to reduce the rate of drop evaporation and thus to minimize the effect of evaporation on the time evolution of monitored parameters.

To test the stability of the superhydrophobic state under contact with water vapor, the sample was kept in the same double wall glass vessel at humidity close to 100% for 24 h. At certain intervals, the sample was removed from the vessel to measure the contact and roll-off angles, and then placed back into water vapor.

The stability of the coating under the joint action of mechanical loads and interaction with water was studied using the cavitation erosion test, which was used as an express test to model the prolonged exposure of coatings to water in conditions of a number of concomitant degrading factors. For that, the samples were exposed to cavitation erosion conditions for a definite time in an ultrasonic water bath with ultrasonic generator frequency of 35 kHz and power of 55 W. The contact and roll-off angles were measured after air-drying the sample for 30 min.

Two methods were used to evaluate the mechanical resistance of the surface texture. In the first method [39] inspired by the ASTM F735 standard [41], the sample was fixed at the bottom of the container, covered with a 25 mm thick layer of calibrated sand (fraction from 500 to 800 µm). The container with sand was placed on the platform of a Vibramax 100 vibration shaker (Heidolph, Germany), which reciprocated with a frequency of 1050 min^−1^ and amplitude of 3 mm. The inertia causes the entire mass of sand to move inside the container and exert a significant abrasive effect on the surface of the sample fixed at the bottom of the container. At certain time intervals, the sample was removed from the container, thoroughly rinsed by distilled water to remove the sand particles embedded into texture and dried for 30 min in oven, and then the contact and roll-off angles were measured to quantify the possible wettability degradation. 

In the second method based on a design described in ASTM D968 standard [42], as implemented by Li et al. [43], a certain amount of sand was released from a height of 20 cm under the action of gravity onto a sample fixed under the sand reservoir at a slope of 45° to the horizon. Calibrated sand, 500 to 800 µm in size, was used. The pouring of a portion of 50 g of sand on the sample was considered as one processing cycle. After each cycle, the sample was washed from sand in an ultrasonic bath at minimum power (35 kHz, 16 W) for 3 min, and then dried in an oven at a temperature of 150 °C for 30 min. After that, the contact and roll-off angles were measured to assess the variation in sample wettability.

The effect of different loads on the texture and chemical composition of surface layers was additionally studied by scanning electron microscopy and energy dispersive X-ray spectroscopy, respectively. Scanning electron microscopy (SEM) images were obtained using a Tescan Amber GMH microscope (Tescan, Czechia). Images were taken at an accelerating voltage of 0.5–20 kV, using an Everhart–Thornley secondary electron detector at a working distance of 4–8 mm and ×750–225,000 magnifications. Energy-dispersive X-ray spectroscopy (EDS) was performed using an Oxford Instruments X-MAX EDS detector at an accelerating voltage of 20 kV and working distance of 6 mm. The EDS detector was calibrated with a cobalt standard. The EDS data were processed using AZtec software.

## 3. Results and Discussion

One of the main requirements for achieving the superhydrophobic state of the coatings on various surfaces is the selection of a treatment regime which ensures the formation of the suitable shape and size of texture elements to provide a heterogeneous wetting regime with a target contact angle. We have studied many samples obtained under various regimes of surface treatment with a laser. Tungsten is one of the most refractory materials; therefore, in all texturing modes, the power was increased, and the processing speed was reduced. Laser texturing of the tungsten surface led to a drop in the contact angle from 58°, characteristic of flat bare metal, to 0° (complete wetting). After the deposition of hydrophobic fluorooxysilane molecules, the surfaces transformed to highly hydrophobic ones (Table 1). Some of these regimes resulted in the superhydrophobic state of the tungsten surface with contact angles above 150° and roll-off angles below 10°. However, as we were seeking regimes which would allow obtaining durable superhydrophobicity, to account for some inevitable degradation under harsh exploitation conditions, we selected for further testing and improving the regimes which led to the highest (e.g., above 170°) contact angles and lowest roll-off angles. Some of the most effective regimes are listed in Table 1 (regimes 3–9) to give an overview of the effects of several varied parameters on the wettability characteristics of obtained coatings. 

Recall that, as stated in the experimental section, in this study, for all samples, the pulse duration of 200 ns and pulse repetition rate of 30 kHz were applied. The effective fluence, *F*, was calculated according to the relation F=NPdf/v, where N is the number of laser passes over each scanned line, P = 0.95 mJ is the single pulse energy, d is the scanning line density, f = 30 kHz is the pulse repetition rate, and v is the speed of the linear movement of the beam during the sample texturing. It is interesting to stress also that rather wide intervals of variation of laser treatment parameters moderately affected the attainable contact angle (the values in the range from 170° to 172° were readily obtained), while the roll-off angles were more sensitive to the particular values of the chosen parameters. 

In the first round of search for a suitable regime, we varied the scanning velocity, which is an important parameter from the point of view of the industrial attractiveness of the method (as it directly affects the time necessary for processing the unit of surface area). It was found that a decrease in scanning velocity (and, hence, an increase in effective fluence) increased the contact angles and reduced the roll-off angles as exemplified in Table 1, regimes 1 to 3. Having in mind the refractoriness of tungsten, we associate such variation in wettability with the effect of surface heating during the laser treatment. 

Therefore, to increase the surface warming up, it was decided to increase the number of repetitive laser passes over the same line (regimes 6 to 8). It is seen that such an increase allowed reducing the droplet pinning, and the roll-off angles fell below 4°. In contrast, the increase in scanning line density (in the case of overlapping scanning lines) weakly affected the wettability parameters (regimes 3 to 5). 

As we were targeted on obtaining the mechanically durable superhydrophobicity, the coatings obtained by regimes 3 to 9 were subjected to the oscillated sand abrasion test. However, already after 20 min of abrasive load, for all tested samples, the contact angles reduced to ~150°, whereas the roll-off angles rose above 65°, thus indicating insufficient durability of the texture. 

Such considerable abrasive wear for the coatings discussed above can be explained as follows. The short duration of an intensive laser pulse and the high velocity of the beam movement along the surface leads to rapid local heating of the surface and its rapid cooling, accompanied by hardening of the surface layer. At the same time, rapid heating of the surface at the applied irradiation power causes explosive ablation with the formation of a surface microrelief and molten metal particles in the laser plume. The subsequent deposition of these particles onto the surface, which is already cooling after the passage of the laser beam, can lead to either the diffusion welding at a high surface temperature or physical deposition of particles bound to the surface by weak van der Waals interactions. In the first case, the adhesion of the deposited micro- and nanoparticles decorating the surface microrelief is strong enough to keep the particles on the surface under external mechanical influences. In the second case, the spontaneous removal of weakly bound particles may occur even upon contact with a water jet or during prolonged immersion in aqueous media. Fast degradation of the coatings obtained by regimes 3–9 indicates the detrimental contribution from the latter mechanism.

To improve the diffusion welding of micro- and nanoparticles re-deposited on the textured surfaces from the laser plume, we combined the laser treatment with a simultaneous additional heating of the samples by placing them on a hot (500 °C) ceramic surface of an electric plate. The laser treatment started after 10 min equilibration, and the electric plate was kept 500 °C hot for the whole duration of the laser treatment. The parameters of the treatment in such a combined regime are listed in Table 2. 

The additional heating did not lead to perceptible variation in the wettability parameters (compare regimes 10–12 with regimes 3–5). To evaluate the adhesion of particles to the surface, the samples were subjected to an oscillating sand abrasive load. As it was expected, the additional, although relatively low, heating of the substrate during its laser texturing allowed achieving a more abrasive resistant coating. It was found that the samples preserve the superhydrophobic state during 20 min of abrasive wear. Moreover, after 2 h abrasion, the samples still remained superhydrophobic, with contact angles reduced to ~160°, and the roll-off angles increased to ~10°. More significant degradation (complete loss of the superhydrophobic state) was observed after 10 h of continuous abrasive treatment. Thus, for sample 10, although the contact angle was still relatively high (154.8±2.2°), the droplets did not roll off from the surface but slid with a sliding angle of 88.1±2.5°, indicating the transition of wettability from heterogeneous (Cassie–Baxter) to homogeneous (Wenzel) mode. Since the best resistance to abrasive loading for 2 h was shown by the sample obtained by regime 10 (contact angle after 2 h abrasion 162.7 ± 0.5°, roll-off angle 9 ± 0.9°), it was chosen as a basis for further improvement of the laser treatment regime.

It was decided to modify regime 10 by increasing the number of laser beam passes to 5. This makes it possible to further increase the laser heating of the tungsten substrate. According to [44], it is reasonable to expect that an increase in a number of passes during texturing which leads to an increase in the substrate temperature, will enhance the bond between surface tungsten atoms and the siloxane groups of fluorooxysilane molecules. After applying this modification (regime 13), the superhydrophobic tungsten surface with a contact angle of 171.5 ± 0.5° and a roll-off angle of 1.5 ± 0.5° was obtained.

**Figure 1:**
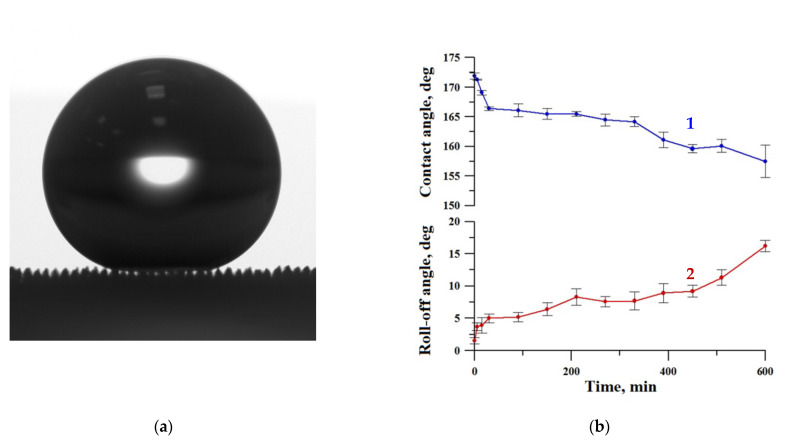
The image of the sessile water droplet (**a**) and the variation of contact (1) and roll-off (2) angles with a time of oscillated sand abrasive load (**b**) for the superhydrophobic coating on tungsten surface obtained by regime 13.

To verify the Cassie–Baxter wetting state of a liquid droplet, the optical image of the sessile droplet on our substrate presented in Figure 1a clearly shows the air captured inside the texture, thus substantiating the heterogeneous wetting regime. This sample was subjected to a more detailed study of mechanical and chemical durability. 

Figure 1 shows the variation of contact and roll-off angles with a time of oscillated sand abrasive load. During the first hour of sand abrasion, the contact angle decreased to ~166° but then the deterioration slowed down, and after 10 h of oscillated abrasive load, the contact angle only reduced to 157° while the roll-off angle increased to 15°. Thus, the sample retained the superhydrophobic properties, although being somewhat degraded.

A similar test for resistance against oscillating sand abrasion was performed recently [45] with superhydrophobic coatings on an aluminum alloy substrate. It was found that after 10 h of abrasive load, the contact angle fell from ~ 173° to ~ 155°, which is comparable to the variation in the contact angle for coatings on a tungsten surface observed in this study. However, the increase in roll-off angle for aluminum substrate (up to 60°) [45] was much higher than for tungsten. Thus, the superhydrophobic coating on the tungsten surface fabricated in this study is more resistant to the detrimental effect of the abrasive load.

To reveal more details about the mechanisms of wettability deterioration and factors counteracting the detrimental effects, we compared the surface morphology and elemental composition of surface layers for samples obtained by regimes 3, 10, and 13. Recall that these samples were processed with the same laser treatment parameters (12.5 lines/mm line scanning density at a linear velocity of 100 mm/s) and only differed by the number of laser passes during surface texturing (single for regimes 3 and 10, and 5-fold for regime 13) and by the assistance of 500 °C hot electric plate heating in regimes 10 and 13.

**Figure 2:**
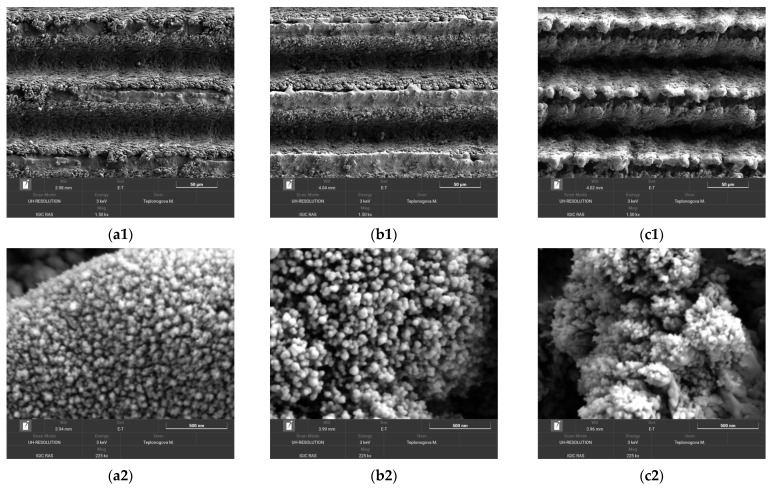
SEM images of tungsten samples after the laser treatment by regimes 3 (images **a1**, **a2**), 10 (**b1**, **b2**), and 13 (**c1**, **c2**) at different magnifications. Scale bars: 50 µm (**a1** to **c1**) and 500 nm (**a2** to **c2**).

Figure 2 shows SEM images of obtained surface textures at two different magnifications. The analysis of images shows that the increase in the temperature at which the laser treatment is performed (electric plate heating in regime 10 versus room temperature treatment in regime 3) leads to an increase in the characteristic size of all important details of the obtained texture of a surface. Thus, the trenches along the laser beam paths are slightly deeper, the sizes of microparticles expelled to the sides of trenches during explosive ablation are larger (compare image b1 against a1 in Figure 2). The nanoparticles redeposited from the laser plume on both the trench wall and the ridges separating the trenches are larger as well (images b2, a2). This is not surprising, as high temperature enhances the diffusion of atoms in micro- and nanoparticles at the texture surface and promotes a kind of Ostwald ripening, that is, a thermodynamically driven spontaneous growth of larger particles at the expense of the reduction of smaller ones. Enhanced diffusion improves the sintering of these particles to the base structures, thus increasing the mechanical durability of obtained texture. All the above arguments equally apply to the next step, the transition from a single-pass laser treatment in regime 10 to 5-fold pass in regime 13. The texture elements clearly enlarged in size and formed pronounced micro-bumps along ridges of texture between deeper trenches (image c1 in Figure 2). At the nanosize level, the growth in particles was not so pronounced, but one can see that the formed structure is looser because of a number of cracks at the surface (image c2).

**Figure 3:**
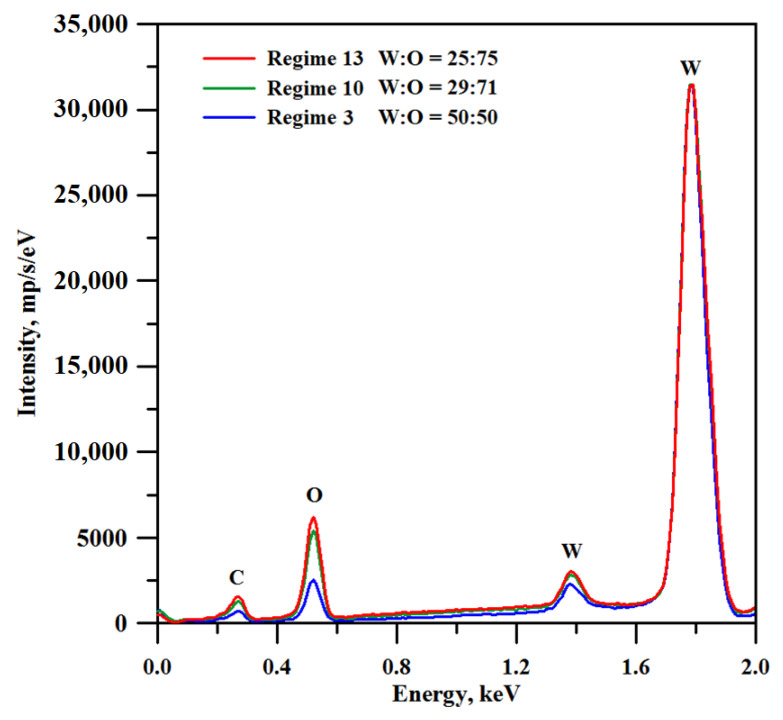
EDS spectra of tungsten samples textured with different laser treatment regimes. Note that the spectra were recorded for the samples prior to hydrophobization.

There is one more factor that should be taken into account when adjusting the treatment parameters for obtaining a suitable texture on the tungsten surface. The increase in the treatment temperature should induce stronger oxidation of tungsten. In Figure 3, we present the EDS spectra for tungsten samples subjected to laser texturing according to discussed regimes. It can be seen from the spectra, and supported by the quantitative estimation (see legend in Figure 3), that the oxygen to tungsten proportion indeed increased with an increase in both the processing temperature and the number of laser beam passes during a surface treatment. However, it is known from the literature data that for non-textured tungsten oxide surface layers, the lower oxygen content corresponds to better resistance to abrasive wear [46]. Therefore, the selection of optimal surface treatment parameters should resolve a trade-off problem between, on the one hand, the improvement of the mechanical durability of the surface texture due to the enlargement of texture elements and enhancement of their diffusion sintering to the base surface, and, on the other hand, the deterioration of the abrasive resistance of surface layers due to the increase in oxygen content. 

**Figure 4:**
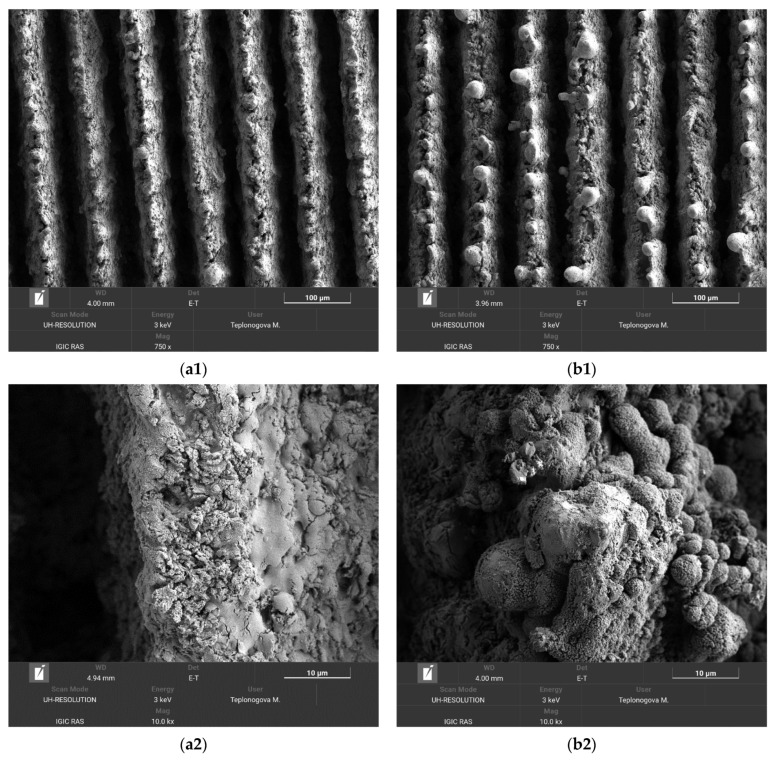
SEM images of the superhydrophobic tungsten sample obtained by regime 13 taken before (**a1**, **a2**) and after (**b1**, **b2**) 10 h of oscillating sand abrasion test. Scale bars: 100 µm (**a1**, **b1**) and 10 µm (**a2**, **b2**).

For the coatings prepared in this study, an additional factor comes into play, related to the specific surface pattern consisting of alternating ridges and trenches. The abrasive wear of top oxide layers on ridges cannot proceed deep into trenches because less oxidized tungsten layers outcropped on ridges after the initial removal of very top layers reveal better abrasive resistance. SEM images presented in Figure 4 well illustrate this point. 

**Figure 5:**
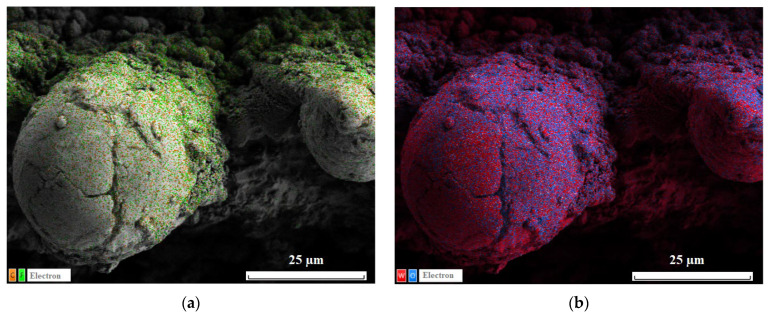
EDS elemental maps for the distribution of (**a**) carbon (orange dots) and fluorine (green); (**b**) tungsten (red) and oxygen (blue) on the surface of superhydrophobic tungsten sample obtained by regime 13 after 10 h of oscillated sand abrasion test.

The comparison of SEM images taken before (a1, a2) and after (b1, b2) 10 h of oscillating sand abrasion test shows that under prolonged abrasive action of moving sand particles the top part of ridges is partially removed, leaving on the surface spheroidal bulges. These bulges, being more stable against abrasion, protect the nanotextures located between them and in deeper trenches promoting the preservation of the superhydrophobic state for the whole coating. Certainly, not only the multimodal roughness but also the low surface energy components of the coating should be preserved to keep the surface superhydrophobic. Figure 5a presents the elemental map showing the surface distribution of carbon and fluorine, the main components of the fluorooxysilane used in this study to decrease the surface energy of the textured tungsten. It is seen that although the tops of bulges protruding above the partially abraded ridges are depleted with fluorooxysilane, the rest of the texture remains evenly covered with hydrophobic components. Following the Cassie rule [47], for a chemically heterogeneous surface, the different patches contribute to the cosine of an effective contact angle proportionally to their portion of the total contact area. Referring to image (b1) in Figure 4, one can ensure that the portion of depleted area is rather small, which explains the moderate degradation of the superhydrophobic properties observed after 10 h oscillated sand abrasion. There is one more feature on the obtained elemental maps worthy of attention. From the distribution of tungsten and oxygen (Figure 5b), one can see brighter red spots on some parts of the bulges. These spots correspond to less oxygen content, and according to the argumentation presented above indicate the places which have better abrasive resistance than more oxidized surrounding parts and thus hamper the further abrasion of the deeper-lying layers of the texture. 

**Figure 6:**
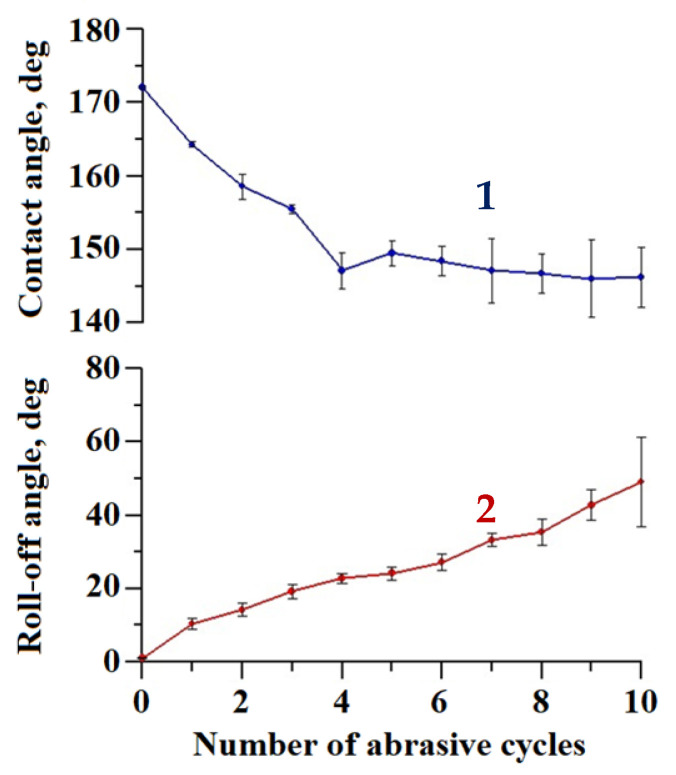
The variation of contact (1) and roll-off (2) angles with a number of falling sand abrasive cycles for the superhydrophobic coating on the tungsten surface obtained by regime 13.

To additionally test the abrasion resistance of the coating, we applied the falling sand method [43], as described above. Compared to the oscillated sand test, this method subjects the surface to a more severe load. On one hand, the tangential (parallel to the sample’s surface) component of the velocity of the sand particles in the falling sand method with parameters used in this work is about 4 times larger than in the oscillating sand method (1.4 m/s versus 0.33 m/s). On the other hand, the falling sand particles exert not only abrasive, but also an impact load on the test surface. This kind of load might be especially detrimental for brittle materials, such as tungsten oxide. Figure 6 presents data on the variation in the wettability during 10 cycles of the falling sand abrasive load.

**Figure 7:**
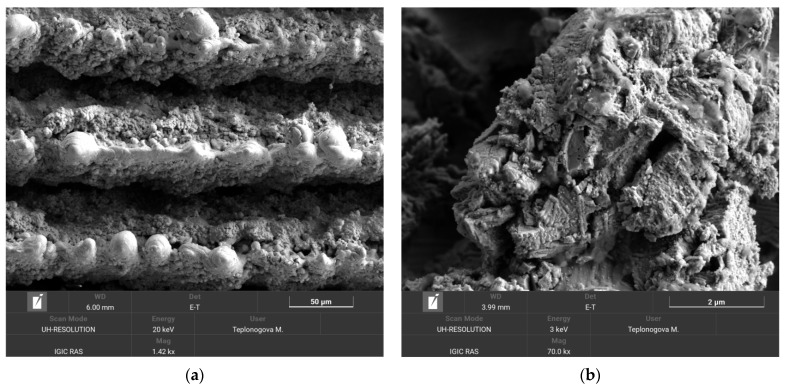
SEM images of the tungsten sample obtained by regime 13 taken after 10 cycles of the falling sand abrasion test. Scale bars: 50 µm (**a**) and 2 µm (**b**).

As expected, in this method, the degradation of the superhydrophobic coating was more pronounced. After 10 loading cycles, the contact angle dropped to 146.2 ± 4.1°, and the roll-off angle increased to 49.0 ± 12.2°. However, the presence of a droplet rolling off indicates that a heterogeneous wetting regime was preserved on the surface. The surface morphology of the sample after the second abrasion test method was studied in order to reveal the distinctive details of surface damage. Figure 7 shows the sample surface morphology after 10 cycles of abrasive loading by falling sand.

Similar to the case of oscillating sand abrasion, it can be seen that some bulges from more abrasive-resistant elements of texture are exposed on top of the ridges. However, the surrounding structures collected more cracks than under oscillating sand, indicating that the developed coating inherited some shortcomings of the brittle base material. Therefore, the intended applications of the developed superhydrophobic tungsten surfaces should avoid significant impact loads during the exploitation.

**Figure 8:**
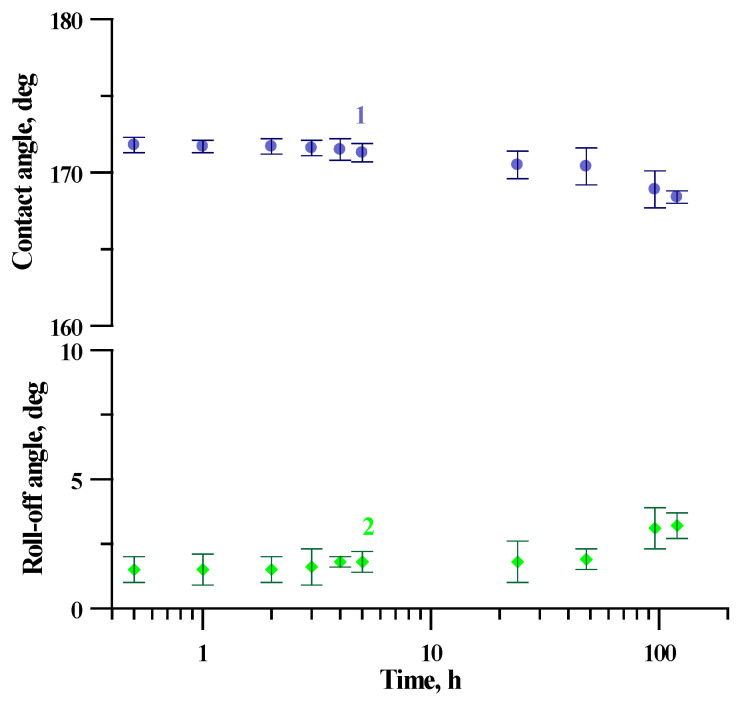
The variation of contact (1) and roll-off (2) angles with the time of exposure to saturated water vapors for the superhydrophobic coating on tungsten surface obtained by regime 13.

The industrial application of tungsten superhydrophobic coatings implies its chemical stability in contact with the water vapors and bulk aqueous phases. The evolution of the superhydrophobic state of fabricated coatings in saturated vapors can be considered one of the most sensitive indicators of the protective properties of a coating against tungsten oxide hydration. To check the protective efficiency of our coating, we studied the variation of their contact and roll-off angles during 5 days of continuous contact with water vapors at relative humidity close to 100% (Figure 8). The very weak evolution of both the contact angle and the roll-off angle with a time of coating exposure to a humid atmosphere allows concluding the good stability of the layer of the fluorooxysilane with respect to the hydrolysis of siloxane bonds in vapors and high protective properties against tungsten oxide hydration.

**Figure 9:**
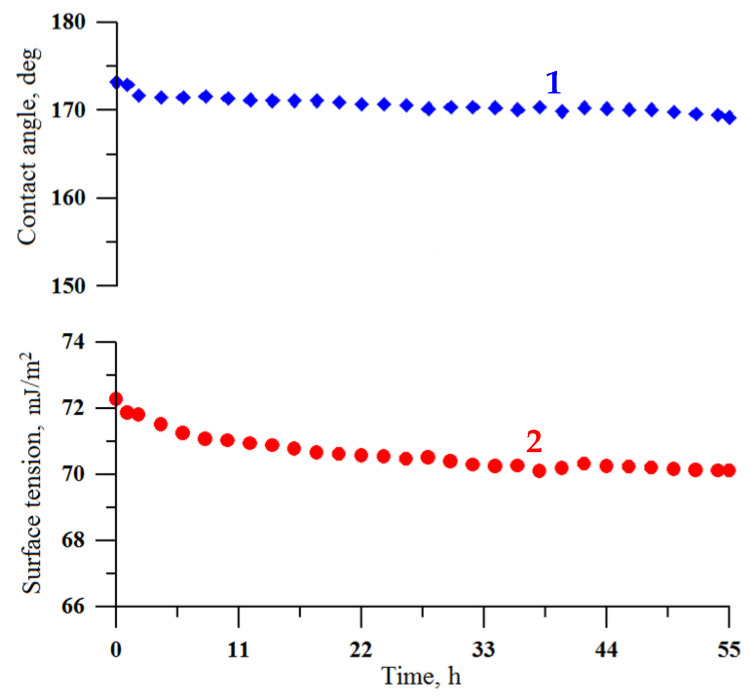
The variation of contact angle (1) and surface tension (2) of a water droplet at prolonged contact with the superhydrophobic coating on the tungsten surface obtained by regime 13.

The chemical durability of the coating in continuous contact with a droplet of water was studied in an atmosphere of saturated water vapors inside a double-wall glass vessel [40]. This experiment allows obtaining information additional to that obtained during the coating exposure to saturated vapor. Namely, in continuous contact with the sessile water droplet, the stability of the fluorooxysilane layer against the hydrolysis-induced desorption of fluorooxysilane molecules from the coating/water interface can be studied. As shown earlier [48,49], the hydrolysis of siloxane bonds in fluorosilanes may cause the desorption of the hydrophobic molecules from the coating, exposing the hydroxyl groups, which are prone to hydration. As a result of this process, the superhydrophobic/hydrophobic state degrades. However, the rate of this process is highly sensitive to the density of fluorosilane molecules on top of the surface layer and their lateral cross-linking [50]. To study the deterioration of the superhydrophobic state for coatings fabricated in this study, the digital images of the droplet were recorded every 10 min and then processed to compute the contact angle and droplet surface tension. Figure 9 shows the variation of contact angle and surface tension of water droplet for almost 60 h of contact. The stability of both reported parameters during long-term continuous contact indicates the formation of a chemically stable layer of hydrophobic fluorooxysilane molecules on the textured tungsten surface.

**Figure 10:**
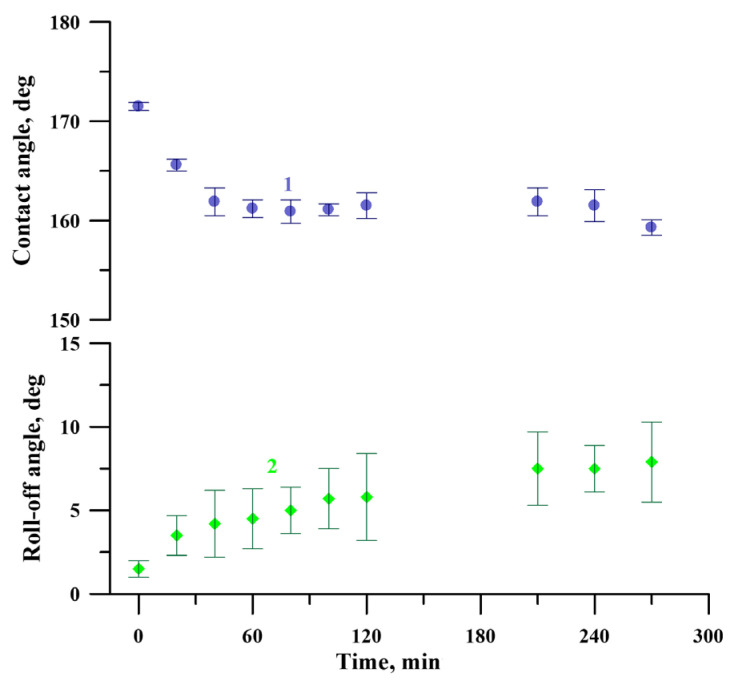
The variation of contact (1) and roll-off (2) angles of a water droplet with the time of the cavitation load for the superhydrophobic coating on the tungsten surface obtained by regime 13.

At the same time, to better characterize the chemical and mechanical stability of the coating in the exploitation conditions, the behavior of the coating was studied using the cavitation erosion test. This type of test can be considered as an express test to estimate the prolonged resistance of the adsorbed layer of fluorooxysilane to hydrolysis in conditions of a number of external degrading factors. Figure 10 shows the evolution of contact and roll-off angles with the time of the cavitation load. Presented data indicate that despite some degradation, the coating preserves the superhydrophobic state, even after 270 min of harsh cavitation loads.

It should be noted that the thermal durability of coatings is another factor playing a major role in the area of applications of coatings. Although tungsten itself is a refractory material, the thermal durability of the superhydrophobic coating is limited by the thermal stability of the hydrophobic agent (fluorooxysilane), used here to decrease the surface energy. As shown earlier [51], such fluorooxysilanes are safely applicable at temperatures *T* ≤ 300 °C.

## 4. Conclusions

Being one of the most refractory materials, tungsten is not easy to work with. Tungsten is prone to oxidation with the formation of oxides having worse mechanical properties than the base metal. Therefore, the elaboration of effective industrially attractive methods for the formation of protective coatings on tungsten surface is highly demanded. In this study, we carefully adjusted the laser treatment parameters and surface hydrophobization regimes and succeeded to fabricate a chemically and mechanically durable superhydrophobic coating on the tungsten surface. The as-prepared coating has a contact angle of 172.1 ± 0.5° and roll-off angle of 1.5 ± 0.4° and preserves high superhydrophobic properties, being subjected to oscillated sand abrasion for 10 h, exposure to saturated water vapors for 24 h, cavitation erosion, continuous contact with water droplets for more than 50 h, and several cycles of the falling sand test.

## Figures and Tables

**Table 1:** The parameters of laser texturing and wettability characteristics of the obtained coatings.

Regime Number	Scanning Velocity v, mm/s	Scanning Line Density d, mm^−1^	Number of Laser Passes N	Effective Fluence F, J/cm^2^	Contact Angle, °	Roll-Off Angle, °
1	500	12.5	1	71	142.1 ± 4.6	No roll-off
2	250	12.5	1	143	147.7 ± 2.6	No roll-off
3	100	12.5	1	356	170.5 ± 0.5	1.6 ± 0.7
4	100	40	1	1140	170.8 ± 0.3	2.2 ± 0.2
5	100	75	1	2138	171.4 ± 0.7	2.1 ± 0.8
6	500	25	1	143	170.2 ± 0.3	7.8 ± 1.8
7	500	25	2	285	171.0 ± 0.5	4.5 ± 1.5
8	500	25	5	715	172.2 ± 0.5	3.5 ± 1.2
9	250	25	1	285	170.3 ± 0.6	7.9 ± 4.5

**Table 2:** The parameters of laser texturing applied in combination with sample heating on the electric plate, and wettability characteristics of the obtained coatings.

Regime Number	Scanning Velocity v, mm/s	Scanning Line Density d, mm^−1^	Number of Laser Passes N	Effective Fluence F *, J/cm^2^	Contact Angle, °	Roll-Off Angle, °
10	100	12.5	1	356	171.3 ± 0.5	1.4 ± 0.5
11	100	40	1	1140	170.1 ± 0.9	3.1 ± 0.6
12	100	75	1	2138	171.2 ± 0.7	2.5 ± 1.1
13	100	12.5	5	1780	171.5 ± 0.5	1.5 ± 0.5

* The fluence was calculated for laser energy only, without accounting for energy gained due to the heating from the electric plate.

## Data Availability

Not applicable.
